# Mental health symptoms and their relations with dietary diversity and nutritional status among mothers of young children in eastern Democratic Republic of the Congo

**DOI:** 10.1186/s12889-019-8092-3

**Published:** 2020-02-13

**Authors:** Jillian A. Emerson, Laura E. Caulfield, Espoir Musafiri Kishimata, Jean-Pierre Nzanzu, Shannon Doocy

**Affiliations:** 1 Department of International Health, Johns Hopkins Bloomberg School of Public Health, 615 N. Wolfe St., Baltimore, MD 21205 USA; 2 Adventist Development and Relief Agency, Uvira, South Kivu Democratic Republic of Congo

**Keywords:** Maternal mental health, Dietary diversity, Maternal nutrition, Sub-Saharan Africa

## Abstract

**Background:**

In developing countries, maternal mental health problems have been linked to sub-optimal child feeding practices and child underweight and stunting, but little is known about how maternal mental health is associated with mothers’ own diets and nutritional status. The objective of the study was to investigate the association between mental health symptoms and diet and nutritional status of mothers of young children in South Kivu, DR Congo.

**Methods:**

Participants were 828 mothers of young children enrolled in a larger, quasi-experimental study evaluating a multi-year food security and nutrition project. The present analysis was conducted with cross-sectional data collected from 2015 to 2016. We assessed symptoms of anxiety and depression using the Hopkins Symptom Checklist-25 (HSCL-25) and post-traumatic stress disorder (PTSD) with the Harvard Trauma Questionnaire (HTQ), using a four-point Likert scale. Mean scale scores were calculated ranging from one to four. A variable was created for high distress (participants scoring in the upper quartile of both measures). Dietary diversity scores were calculated from the number of food groups (range zero to ten) consumed the previous day, identified from an open recall. Nutritional status was measured by body mass index (BMI) and underweight (BMI < 18.5 kg/m^2^, or mid-upper arm circumference < 23 cm for pregnant women). Bivariate and multivariate (adjusting for parent study intervention group, education, age, health, parity, livelihoods zone, and territory of origin) regression analyses were conducted.

**Results:**

Maternal mental health measures were positively and statistically significantly associated with higher dietary diversity scores in adjusted analyses (HSCL-25: ß= 0.18, *p* = 0.002, HTQ: ß= 0.12, *p* = 0.029, High Distress: ß= 0.47, *p* < 0.001). Mental health symptoms were not significantly associated with BMI (HSCL-25: ß = − 0.04, *p* = 0.824; HTQ: ß = 0.02, *p* = 0.913; High distress: ß= − 0.02, *p* = 0.938) or underweight (HSCL 25: OR = 0.91, *p* = 0.640; HTQ: OR = 1.03, *p* = 0.866; High distress: OR = 0.78, *p* = 0.489).

**Conclusions:**

More severe maternal mental health symptoms were associated with higher dietary diversity but not nutritional status, and the reasons for these findings are not clear from available data. More research is needed to identify underlying factors that could influence mental health symptomatology and diet quality among food insecure and extremely resource-limited populations.

## Background

Mental health problems are common morbidities in the perinatal period, and are associated with a wide range of adverse health outcomes, including preterm birth and child stunting and underweight [1, 2]. There exists a great deal of evidence from low-income countries on the link between maternal depression and child undernutrition. Depression symptoms, such as apathy and sadness, may impede mothers’ ability to care for and feed infants and young children optimally, contributing to poor health and nutrition outcomes [3–5]. In developing countries, it is estimated that about 15% of pregnant women experience a mental health disorder in the antenatal period and 20% experience a postnatal mental disorder, which is higher than the estimates of 10 and 13% respectively in high-income countries [1]. At the same time, women in developing countries are especially vulnerable to undernutrition, notably in the perinatal period when pregnancy and lactation increase nutritional requirements.

In sub-Saharan Africa, many studies have focused on links between mental health and food insecurity, but there has been little research on maternal mental health and maternal nutrition [6–8]. Evidence from high-income countries indicates that mental health problems among perinatal women are associated with disordered eating attitudes, higher body mass index (BMI), increased risk for overweight/obesity, and lower consumption of micronutrients [9–11]. Among adults in high-income countries, depressive symptoms are associated with decreased consumption of fruits and vegetables and increased consumption of energy-dense sweet foods [12, 13]. Conversely, high fruit and vegetable intake is associated with lower risk of depression [14], and the Mediterranean diet, which is high in B vitamins and polyunsaturated fatty acids (PUFAs), has also be found to reduce depression risk [15, 16]. Higher concentrations of omega-3 PUFAs in breast milk are associated with decreased risk for depression and several studies have found improvements in depression symptoms with omega-3 PUFA supplementation [17–19]. Specific micronutrient deficiencies are associated with symptoms of mental health problems, such as zinc, which is important for neurotransmitter functioning. Lower plasma or serum zinc concentrations are associated with depression symptoms [20]. Folate and Vitamin B-12 may play a role in the synthesis and metabolism of neurotransmitters, and deficiencies may cause elevated concentrations of homocysteine, which are related to depressed mood [21, 22]. Symptoms of iron-deficiency, including fatigue, irritability, and apathy, overlap with depression symptoms [23].

Yet there has been little focus on understanding how maternal mental health problems are associated with maternal diet and nutritional status in developing countries, even though poverty and women’s disempowerment are risk factors for both mental health problems and undernutrition [24–26]. The objective of this study is to evaluate the association between symptoms of mental health problems and the diet and nutritional status of mothers of young children in South Kivu province, in eastern Democratic Republic of Congo (DRC). The DRC’s eastern provinces have experienced years of armed conflict, population displacement, and human rights violations which have had a devastating impact on the food security, nutrition, and mental health status of the population [27]. Lack of infrastructure, rising food prices, poverty, and a poorly functioning health system have contributed to alarming rates of chronic undernutrition [28]. Over half of children under five in South Kivu province are stunted (53%), higher than the national prevalence of 43% [29]. A survey conducted in North and South Kivu found that 50% of the population had symptom criteria for post-traumatic stress disorder (PTSD) and 40% for major depression [27]. We hypothesized that in this highly food insecure context, higher symptoms of maternal mental health problems are associated with lower dietary diversity, lower BMI, and increased odds of maternal underweight.

## Methods

### Parent study

The present study is a sub-study of a larger, quasi-experimental evaluation of a United States Agency for International Development (USAID) food assistance program called Jenga Jamaa II [30]. Jenga Jamaa II was designed to improve household food security and child nutrition in Uvira and Fizi territories in South Kivu through four distinct nutrition-specific and nutrition-sensitive interventions, and was implemented by the non-governmental organizations (NGOs) Adventist Development and Relief Agency (ADRA) and World Vision International from 2011 to 2016. Enrollment of project beneficiaries and control group participants for the parent study occurred from August to October 2012, with 1820 households enrolled and followed for three and a half years. Data were collected via eight cross-sectional surveys occurring in August/September and February/March of each year to account for seasonal variation in food security. More details related to the parent study can be found elsewhere [30].

### Data collection

The present study is a cross-sectional sub-study utilizing data collected during the last two Jenga Jamaa II surveys conducted in September 2015 and March 2016. Participants from the Jenga Jamaa II parent study who were mothers of children under five also enrolled were eligible for the sub-study. Individuals were excluded from the study if they were not enrolled in the parent study or were not the biological mother of a child also enrolled in the parent study. Maternal mental health was assessed at one time point (September 2015 for the majority of the participants), but maternal diet and weight data were utilized from both the September 2015 and March 2016 surveys (among participants present at both) to account for seasonal variation. Data were collected electronically using the mobile data collection application Magpi and Android tablets provided by ADRA [31].

The Institutional Review Board of the Johns Hopkins Bloomberg School of Public Health approved all data collection instruments and procedures. At every data collection encounter for the parent study, study staff obtained oral consent in Swahili, the predominant local language, and participants were reminded that they could decline to participate at any time. Additional oral consent was requested for participation in the sub-study, in which the mental health questionnaire was described as well as the potential risks and benefits of participation. ADRA field agents who served as study enumerators received training on the questionnaire and on research ethics prior to data collection.

### Measures

#### Independent variables

Independent variables assessed were depression/anxiety symptoms, post-traumatic stress disorder (PTSD) symptoms, and a variable (high mental distress) constructed to identify participants with high levels of both depression/anxiety and PTSD symptoms, having mean item scale scores in the upper quartile of both measures. The Hopkins Symptom Checklist (HSCL-25) was used to assess depression symptoms (15 items) and anxiety symptoms (10 items), and the Harvard Trauma Questionnaire (HTQ) was used to assess PTSD symptoms (16 items) [32, 33]. One item on suicidality was dropped from the HSCL-25 due to ethical considerations. Participants rated the frequency of each symptom in the prior four weeks on a four-point Likert scale, and mean item scores were calculated for depression/anxiety (HSCL-25) and PTSD (HTQ), with a possible range of one to four.

The mental health questionnaire that included the HSCL-25 and HTQ tools was adapted from an earlier study in South Kivu that evaluated the impact of a cognitive behavioral therapy intervention among female survivors of sexual violence [34]. The questionnaire was administered in Swahili, the predominant local language. The HSCL-25 is frequently used in a variety of cultural settings [7, 35–37], and the validity of a Swahili version of the scale has been evaluated in a sample of Tanzanian women using content and construct validation methods [36]. Additionally, it has been used to assess depression symptoms among Congolese refugees in the United States [38]. The validity of the Harvard Trauma Questionnaire has been assessed in multiple settings and is often used among populations who have experienced conflict and displacement, such as refugees [33, 39–41].

After data collection was complete, the internal consistency reliability of mental health measures was assessed using Cronbach’s alpha [42]. The HSCL-25 items had a scale reliability coefficient of 0.92 and the HTQ items had a scale reliability coefficient of 0.91. The anxiety and depression subscales of the HSCL-25 had a correlation coefficient of 0.72 (*r*(826), *p* < 0.001). The correlation coefficient of the HSCL-25 and HTQ was 0.82 (*r*(826), *p* < 0.001).

#### Dependent variables

Maternal BMI and underweight were used as measures of maternal nutritional status. Maternal height, weight, and mid-upper arm circumference (MUAC) were measured by trained ADRA field agents using standard protocols [43]. BMI was calculated for non-pregnant participants, and MUAC was used to assess nutritional status of pregnant participants [44]. Weight was averaged for the two data collection periods if participants were present at both surveys, or a sole weight measure was used for participants who were only present at one survey. Pregnant mothers with MUAC < 23 cm and non-pregnant mothers with BMI < 18.5 kg/m^2^ were classified as underweight [44]. Participants were measured using a Model 1582 Tanita Mommy and Baby Infant Scale (Arlington Heights, IL) and a Shorr Productions (Olney, MD) height board.

Dietary diversity scores were used to measure diet quality, using the Minimum Dietary Diversity for Women (MDD-W) tool [45]. Enumerators asked participants to list all foods consumed the previous day and night. When composite dishes were mentioned, they asked for a list of ingredients and probed for additional items. All of the food items were recorded in Swahili, and then translated to English and classified into one of ten possible food groups: 1) starchy staples (grains, white roots, tubers, and plantains); 2) pulses (beans, peas, lentils); 3) nuts and seeds; 4) dairy; 5) meat, poultry, and fish; 6) eggs; 7) dark green leafy vegetables; 8) other vitamin A-rich fruits and vegetables; 9) other vegetables; and 10) other fruit. The number of food groups consumed was summed to create a dietary diversity score, with a possible range of zero to ten, with higher scores indicating greater diversity. Maternal dietary data were collected at two time points six months apart, and an average score was calculated, in order to address seasonal variation.

#### Background and demographic variables

The mental health questionnaire included a section on background and demographic characteristics: age, years of education obtained, ethnic group, living in territory of origin, currently pregnant, number of children, and marital status. Education was recoded as a categorical variable with three categories: no education, completing at least some primary school, and completing at least some secondary school or higher education. Participants were also asked if they had a child that died, and to rate their physical health status, using a scale that ranged from excellent to poor.

Household-level data, including household size, income in the past month, and food insecurity, were collected as part of the parent study survey questionnaire. Food insecurity was measured using the Household Food Insecurity Access Scale (HFIAS) [46]. Households were classified in categories ranging from food secure to severely food insecure based on HFIAS score. Participants were also asked their income in the past month. Household income was not included in the final analysis because it was not necessarily reflective of socioeconomic status; in this context it may have represented the sale of assets due to hardship or food insecurity. Indicator variables for intervention groups were created for the four parent study interventions and the control group. Indicator variables for geographic region (Uvira or Fizi territory) and livelihoods zone (plains, mountains, or lakeside) were also included.

### Statistical analysis

Data were analyzed using Stata 13 [47]. Distributions of continuous variables and frequencies of categorical variables were explored, and outlying values were identified. The three dependent variables assessed were dietary diversity score, BMI, and underweight. Dietary diversity score was a continuous variable. BMI was continuous and limited to non-pregnant participants. Underweight was constructed as a binary variable. Independent variables for maternal mental health were mean item HSCL-25 score (measuring depression and anxiety symptoms), mean item HTQ score (measuring PTSD symptoms), and a binary variable for high psychological distress (upper quartile of both measures). Separate analyses were conducted for each of the three independent variables due to multicollinearity.

Bivariate regression analyses were conducted between demographic/socioeconomic measures and independent and dependent variables (Additional file 1: Tables 4 and 5). Potential confounding variables were selected for inclusion in the model if they were associated with both independent and dependent variables, or if they had a conceptual relationship to both. Multivariate linear and logistic regression analyses were conducted, adjusting for potential confounding variables.

## Results

The final sample included 828 participants after the exclusion of data from 40 participants whose children were not enrolled in the parent study, four who were not the biological mothers of the children enrolled, and six who did not have data recorded for any dependent variable.

### Socio-demographic characteristics of the sample

Demographic and background characteristics of the sample are presented in Table 1. The mean age of the participants was 29.6 years, and almost all (90.1%) were married. About one-third of participants (31.7%) had never attended school, 48.7% had some primary school education, and 19.7% had at least some secondary school education. The participants had an average of five children (range: 1–11), and 28.9% had at least one child that died. About 20% reported they were pregnant at the time of the survey. The average household size was 7.8 people, and median household income in the previous month was 22,500 Congolese francs (approximately $23). The majority of households (67.8%) were classified as severely food insecure.

**Table 1:** Background Characteristics of Study Sample, *N* = 828

	Mean (SD) or N (%)
Territory	
Uvira	440 (53.1)
Fizi	388 (46.5)
Livelihoods Zone	
Mountains	114 (13.7)
Plains	459 (55.4)
Lakeside	255 (30.6)
Intervention Group^a^	
WEG	152 (18.4)
PM2A	285 (34.4)
FFS	118 (14.3)
F2F	113 (13.6)
Control	160 (19.3)
Household Size	7.8 (2.6)
Household Income, Median (IQR)^b^	22,500 (9500, 45,000)
Severely Food Insecure^c^	556 (67.8)
Maternal Age	29.6 (6.3)
Education Level	
None	261 (31.7)
Some Primary School	401 (48.7)
Some Secondary School	162 (19.7)
Married	745 (90.1)
Ethnic Group	
Mubembe	350 (42.4)
Mufuliro	396 (47.9)
Murundi	16 (1.9)
Mushi	26 (3.1)
Muvira	11 (1.3)
Other	27 (3.3)
Living in Territory of Origin	618 (74.7)
Self-reported Health Status	
Poor	221 (26.9)
Average	298 (36.2)
Good	270 (32.8)
Very good/Excellent	34 (4.1)
Number of Children	5.4 (2.3)
Experienced Death of a Child	239 (28.9)
Pregnant	160 (19.3)

^a^*WEG* Women’s Empowerment Group, *PM2A* Prevention of Malnutrition in Children under 2 Approach, *FFS* Farmer Field Schools, *F2F* Farmer to Farmer

^b^Past month, Congolese francs

^c^Household Food Insecurity Access Scale

### Maternal mental health, diet, and nutritional status descriptive statistics

Descriptive statistics for independent and dependent variables are presented in Table 2. Participants’ average mean item scores on the mental health measures were 2.3 on the HSCL-25 and 2.1 on the HTQ. There were 142 participants (17.2%) whose mean item scores were in the upper quartile of both measures and were classified as having high psychological distress. Only three participants, or less than 1% of the sample, had mean item scores of one on the HSCL-25, indicating they had experienced no depression or anxiety symptoms in the past four weeks, and 3% had experienced no PTSD symptoms.

**Table 2:** Maternal Mental Health, Dietary Diversity, and Nutritional Status Descriptive Statistics

		Mean (SD) or N (%)
Maternal Mental Health (*n* = 828)		
HSCL-25^a^		2.3 (0.6)
HTQ^b^		2.1 (0.6)
High Distress^c^		142 (17.2)
Maternal Nutritional Status (*n* = 782)		
Weight (kg)^d^		
Survey 1		50.9 (8.06)
Survey 2		49.5 (7.7)
Average, Both Surveys		50.3 (7.6)
Height (m)		1.5 (0.1)
BMI (kg/m^2^)^d^		21.8 (2.8)
MUAC (cm) (Pregnant participants)		25.2 (2.7)
Underweight^e^		86 (11.0)
Maternal Diet (*n* = 804)		
Dietary Diversity Score		
Survey 1		2.5 (1.2)
Survey 2		2.8 (1.1)
Average, Both Surveys		2.6 (1.0)

^a^24 items from the Hopkins Symptom Checklist-25 measuring depression and anxiety symptoms, range of scale scores 1–4.

^b^16 items from the Harvard Trauma Questionnaire measuring post-traumatic stress symptoms, range of scale scores 1–4.

^c^Participants with mean item scores in the upper quartile of both measures (≥ 2.6 for the HSCL-25 and ≥ 2.5 for the HTQ).

^d^Among 634 non-pregnant participants.

^e^BMI < 18 kg/m^2^ among non-pregnant participants or MUAC < 23 cm among pregnant participants.

Maternal dietary data were available from 804 participants (97.1%), of which 507 were present for both surveys. The mean dietary diversity score was 2.5 during the first survey (*n* = 730, range 0–7) and the mean score was 2.8 for the second survey (*n* = 581, range 0–6). The average mean dietary diversity score was 2.6 (Table 2). Only 3% of the participants achieved the Minimum Dietary Diversity for Women indicator (consuming at least five of ten food groups in the previous day) [45].

Participants' diets mostly consisted of *fufu*, a fluffy starch made from either cassava or corn flour, and *sombé* (cassava leaves). Few mothers consumed animal source foods or legumes. In addition to cassava leaves, other dark green leafy vegetables consumed included sweet potato leaves, bean leaves, and *lenga-lenga* (amaranth greens). Consumption of other vitamin A–rich fruits or vegetables was rare. Those with higher dietary diversity scores tended to consume more fruits and vegetables, including tomato, avocado, eggplant, zucchini, mushroom, onion, mango, and guava. Very few participants consumed eggs or dairy.

Anthropometric data were available from 782 (94.4%) participants, and descriptive statistics are displayed in Table 2. Weight was collected during both surveys and averaged to account for seasonality. Mean weight during the second survey was slightly lower (49.5 kg) compared to the first survey (50.9 kg), and mean height was 1.5 m. Mean BMI among non-pregnant participants was 21.8 kg/m^2^, and 8.4% were underweight. Mean MUAC of the 194 pregnant participants was 25.2 cm, and 22.3% were classified as underweight. In total, 11% of pregnant and non-pregnant participants combined were underweight.

### Bivariate analyses

Results of bivariate and multivariate analyses are presented in Table 3. In crude models, there was a positive association between higher mental health symptoms and dietary diversity score, with every 1-point increase in HSCL-25 score associated with an increase of 0.22 in dietary diversity (*p* < 0.001). Similar results were found when assessing the HTQ score (β = 0.15, *p* = 0.004), and high psychological distress (β = 0.48, *p* < 0.001). A negative but not statistically significant association was found between maternal mental health variables and BMI. Unadjusted beta coefficients were − 0.17 for HSCL-25 (*p* = 0.355), − 0.03 for HTQ (*p* = 0.869), and − 0.19 for high psychological distress (*p* = 0.512). No significant associations were found between maternal mental health variables and odds of underweight (HSCL-25: OR = 1.01, *p* = 0.967, HTQ: OR = 1.07, *p* = 0.722, High Distress: OR = 0.84, *p* = 0.577).

**Table 3:** Bivariate and Multivariate Associations between Maternal Mental Health Symptoms, Dietary Diversity and Nutritional Status

Dietary Diversity Score (*n* = 776)						
	Crude			Adjusted^a^		
	β	(95% CI)	*p*-value	β	(95% CI)	*p*-value
HSCL-25^b^	0.22	(0.10, 0.33)	**<0.001**	0.18	(0.06, 0.30)	**0.002**
HTQ^c^	0.15	(0.05, 0.26)	**0.004**	0.12	(0.01, 0.23)	**0.029**
High Distress^d^	0.48	(0.31, 0.66)	**<0.001**	0.47	(0.28, 0.65)	**<0.001**
BMI (*n* = 616)						
	Crude			Adjusted^a^		
	β	(95% CI)	*p*-value	β	(95% CI)	*p*-value
HSCL-25^b^	-0.17	(-0.53, 0.19)	0.355	-0.04	(-0.42, 0.33)	0.824
HTQ^c^	-0.03	(-0.36, 0.30)	0.869	0.02	(-0.32, 0.36)	0.913
High Distress^d^	-0.19	(-0.75, 0.37)	0.512	-0.02	(-0.61, 0.56)	0.938
Underweight (*n* = 761)						
	Crude			Adjusted^a^		
	OR	(95% CI)	*p*-value	OR	(95% CI)	*p*-value
HSCL-25^b^	1.01	(0.69, 1.46)	0.967	0.91	(0.60, 1.37)	0.640
HTQ^c^	1.07	(0.75, 1.51)	0.722	1.03	(0.71, 1.51)	0.866
High Distress^d^	0.84	(0.45, 1.56)	0.577	0.78	(0.40, 1.56)	0.489

^a^Adjusted for intervention group, age, education level, living in territory of origin, livelihoods zone, number of children, and self-reported health status

^b^24 items from the Hopkins Symptom Checklist-25 measuring depression and anxiety symptoms, range of scale scores 1–4

^c^16 items from the Harvard Trauma Questionnaire measuring post-traumatic stress symptoms, range of scale scores 1–4

^d^Participants with mean item scores in the upper quartile of both measures (≥ 2.6 for the HSCL-25 and ≥ 2.5 for the HTQ)

Significant *p*-values are set in bold

### Multivariate analyses

Multivariate analyses adjusted for covariates identified as potential confounding variables: intervention group, age, education level, number of children, living in territory of origin, livelihoods zone, and self-reported health status. In adjusted analyses (Table 3), positive and statistically significant relations were found between HSCL-25 (β = 0.18, *p* = 0.002), HTQ (β = 0.12, *p* = 0.029), and high psychological distress (β = 0.47, *p* < 0.001) and dietary diversity. In the adjusted model with BMI as a dependent variable, no significant associations were found. Beta coefficients were − 0.04 for HSCL-25 (*p* = 0.824), 0.02 for HTQ (*p* = 0.913) and − 0.02 for high psychological distress (*p* = 0.938). No association was found between maternal mental health and odds of underweight (HSCL 25: OR = 0.91, *p* = 0.640, HTQ: OR = 01.03, *p* = 0.866, High distress: OR = 0.78, *p* = 0.489).

## Discussion

This study found that mothers of young children with higher levels of depression, anxiety, and PTSD symptoms had higher dietary diversity scores, with every one-unit increase in mean item scale scores associated with small but statistically significant increases in dietary diversity scores. Regardless of mental health status, all participants had low dietary diversity, consuming on average less than three food groups the previous day. No associations were found between maternal mental health symptoms and BMI or odds of underweight. These findings indicate that mothers with more severe mental health symptoms had slightly more diverse and thus higher quality diets compared to mothers with less severe symptoms, and that maternal mental health symptoms were not related to nutritional status. An explanation for the positive relations between maternal mental health and dietary diversity was not clear from available data.

The extremely resource-limited context may have been an important factor contributing to the contrary findings. The results of the parent study found that stunting prevalence ranged from 55 to 70% among children in intervention group households and 47% of households were severely food insecure at endline, making this a particularly nutritionally deprived group [30, 48]. In such a food insecure environment, opportunities to improve dietary diversity are limited, and differences in dietary diversity among participants with higher levels of mental health symptoms compared to those with less severe symptoms may be due to other underlying or modifying factors, such as social support and work burden. For example, social support was found to modify the association between depression and food insecurity among people living with HIV/AIDS in Uganda, as well as among perinatal women in urban South Africa [49, 50]. Additionally, women who have a higher work burden may experience increased stress and related mental health symptoms but might also have more income with which to purchase more diverse foods. However, it is not possible to assess these factors in the context of the present study.

Our finding that maternal mental health was positively associated with dietary diversity differs from other studies’ findings, although there are few comparable studies on this topic from other developing countries, nor from post-conflict, severely food insecure settings. A recent systematic review found that perinatal depression and stress symptoms were associated with less healthy diets, and another found that high intakes of micronutrient-rich foods such as vegetables and fruit were associated with lower depression risk among adults [51, 52]. One study in Iran assessing only anxiety symptoms found that they were associated with low dietary diversity among women [53].

With regard to maternal mental health symptoms and maternal nutritional status, our finding that there was no association between more severe symptoms and BMI also diverged from the existing literature. Studies from high-income countries found relations between depressive symptoms and BMI to resemble a U-shaped curve, where depressive symptoms are associated with both underweight and overweight in adults [54–56]. Among women in the perinatal period, one study found that women with normal pre-pregnancy BMI (compared to obese and underweight women) had signficantly lower risk of postpartum depression symptoms.

To our knowledge, this is the first study to assess maternal mental health in relation to maternal dietary diversity and nutritional status in sub-Saharan Africa. The study evaluated symptoms of multiple mental health problems in the same population, utilizing measures which have been found to be valid among similar ethno-linguistic groups and conflict-affected populations. A limitation of the study was that a number of factors limit the generalizability of the findings, including that the majority of the participants were enrolled in a food security/nutrition intervention, and that most households were severely food insecure (despite the intervention). Additionally, mental health symptoms and diet were self-reported, thus recall bias may have affected the validity of the findings. Other studies have shown that major depression is associated with memory bias [57, 58], therefore participants experiencing higher levels of mental health systems may have been particularly susceptible to bias. Finally, the study is cross-sectional, so it is not possible to understand directionality of the findings.

## Conclusions

Our study found that higher levels of maternal mental health symptoms were associated with higher maternal dietary diversity scores, but not associated with maternal BMI or underweight. The findings suggest that despite the presence of large-scale food security and nutrition interventions, food insecurity and undernutrition remained prevalent, and any improvements in household food security as a result of the interventions may not have necessarily extended to women’s diets. Symptoms of depression, anxiety, and post-traumatic stress were highly prevalent among the study sample. Regardless of our findings, programs seeking to improve the well-being of vulnerable populations in similar contexts might consider focusing on maternal mental health and engaging in more intensive efforts to improve maternal diet quality.

## Supplementary information

**Additional file 1: Table 4.** Associations between Independent Variables and Background Characteristics. **Table 5.** Associations between Dependent Variables and Background Characteristics.

## Data Availability

The data that support the findings of this study are available on request from the corresponding author JAE. The data are not publicly available due to them containing information that could compromise research participant privacy.

## Abbreviations

ADRA: Adventist Development and Relief Agency
BMI: Body mass index
DHS: Demographic and Health Survey
F2F: Farmer-to-Farmer
FFS: Farmer Field Schools
HFIAS: Household Food Insecurity Access Scale
HSCL-25: Hopkins Symptom Checklist-25
HTQ: Harvard Trauma Questionnaire
MDD-W: Minimum Dietary Diversity for Women
MUAC: Mid-upper arm circumference
NGO: Non-governmental organizations
PM2A: Preventing Malnutrition in Children under 2 Approach
PTSD: Post-traumatic stress disorder
PUFA: Polyunsaturated fatty acid
USAID: United States Agency for International Development
WEG: Women’s Empowerment Group

## References

[1] Fisher J, Cabral de Mello M, Patel V, Rahman A, Tran T, Holton S (2012). Prevalence and determinants of common perinatal mental disorders in women in low- and lower-middle-income countries: a systematic review. Bull World Health Organ.

[2] Howard LM, Molyneaux E, Dennis CL, Rochat T, Stein A, Milgrom J (2014). Non-psychotic mental disorders in the perinatal period. Lancet.

[3] Prince M, Patel V, Saxena S, Maj M, Maselko J, Phillips MR (2007). No health without mental health. Lancet.

[4] Rahman A, Patel V, Maselko J, Kirkwood B (2008). The neglected 'm' in MCH programmes - why mental health of mothers is important for child nutrition. Tropical Med Int Health.

[5] Rahman A, Surkan PJ, Cayetano CE, Rwagatare P, Dickson KE (2013). Grand challenges: integrating maternal mental health into maternal and child health programmes. PLoS Med.

[6] Dewing S, Tomlinson M, le Roux IM, Chopra M, Tsai AC (2013). Food insecurity and its association with co-occurring postnatal depression, hazardous drinking, and suicidality among women in peri-urban South Africa. J Affect Disord.

[7] Hadley C, Tegegn A, Tessema F, Cowan JA, Asefa M, Galea S (2008). Food insecurity, stressful life events and symptoms of anxiety and depression in East Africa: evidence from the Gilgel gibe growth and development study. J Epidemiol Community Health.

[8] Sorsdahl K, Slopen N, Siefert K, Seedat S, Stein DJ, Williams DR (2011). Household food insufficiency and mental health in South Africa. J Epidemiol Community Health.

[9] Hurley KM, Caulfield LE, Sacco LM, Costigan KA, Dipietro JA (2005). Psychosocial influences in dietary patterns during pregnancy. J Am Diet Assoc.

[10] Senturk V, Hanlon C, Medhin G, Dewey M, Araya M, Alem A (2012). Impact of perinatal somatic and common mental disorder symptoms on functioning in Ethiopian women: the P-MaMiE population-based cohort study. J Affect Disord.

[11] Emerson JA, Hurley KM, Caulfield LE, Black MM. Maternal mental health symptoms are positively related to emotional and restrained eating attitudes in a statewide sample of mothers participating in a supplemental nutrition program for women, infants and young children. Matern Child Nutr. 2017;13:e12247. 10.1111/mcn.12247 PMC6866000 26898604

[12] Konttinen H, Mannisto S, Sarlio-Lahteenkorva S, Haukkala A (2010). Emotional eating, depressive symptoms and self-reported food consumption. A population-based study. Appetite.

[13] Jeffery RW, Linde JA, Simon GE, Ludman EJ, Rohde P, Ichikawa LE (2009). Reported food choices in older women in relation to body mass index and depressive symptoms. Appetite.

[14] Saghafian F, Malmir H, Saneei P, Milajerdi A, Larijani B, Esmaillzadeh A (2018). Fruit and vegetable consumption and risk of depression: accumulative evidence from an updated systematic review and meta-analysis of epidemiological studies. Br J Nutr.

[15] Sanchez-Villegas A, Martinez-Gonzalez MA, Estruch R, Salas-Salvado J, Corella D, Covas MI (2013). Mediterranean dietary pattern and depression: the PREDIMED randomized trial. BMC Med.

[16] Parletta N, Zarnowiecki D, Cho J, Wilson A, Bogomolova S, Villani A (2019). A Mediterranean-style dietary intervention supplemented with fish oil improves diet quality and mental health in people with depression: a randomized controlled trial (HELFIMED). Nutr Neurosci.

[17] Hibbeln JR (2002). Seafood consumption, the DHA content of mothers' milk and prevalence rates of postpartum depression: a cross-national, ecological analysis. J Affect Disord.

[18] Peet M, Horrobin DF (2002). A dose-ranging study of the effects of ethyl-eicosapentaenoate in patients with ongoing depression despite apparently adequate treatment with standard drugs. Arch Gen Psychiatry.

[19] Nemets B, Stahl Z, Belmaker RH (2002). Addition of omega-3 fatty acid to maintenance medication treatment for recurrent unipolar depressive disorder. Am J Psychiatry.

[20] DiGirolamo AM, Ramirez-Zea M (2009). Role of zinc in maternal and child mental health. Am J Clin Nutr.

[21] Folstein M, Liu T, Peter I, Buell J, Arsenault L, Scott T (2007). The homocysteine hypothesis of depression. Am J Psychiatry.

[22] Bodnar LM, Wisner KL (2005). Nutrition and depression: implications for improving mental health among childbearing-aged women. Biol Psychiatry.

[23] Murray-Kolb LE, Beard JL (2009). Iron deficiency and child and maternal health. Am J Clin Nutr.

[24] Kinyanda E, Woodburn P, Tugumisirize J, Kagugube J, Ndyanabangi S, Patel V (2011). Poverty, life events and the risk for depression in Uganda. Soc Psychiatry Psychiatr Epidemiol.

[25] Garcia ER, Yim IS (2017). A systematic review of concepts related to women's empowerment in the perinatal period and their associations with perinatal depressive symptoms and premature birth. BMC Pregnancy Childbirth.

[26] Ruel MT, Alderman H (2013). Maternal, child nutrition study G. nutrition-sensitive interventions and programmes: how can they help to accelerate progress in improving maternal and child nutrition?. Lancet.

[27] Johnson K, Scott J, Rughita B, Kisielewski M, Asher J, Ong R (2010). Association of sexual violence and human rights violations with physical and mental health in territories of the eastern Democratic Republic of the Congo. JAMA.

[28] Kandala NB, Madungu TP, Emina JB, Nzita KP, Cappuccio FP (2011). Malnutrition among children under the age of five in the Democratic Republic of Congo (DRC): does geographic location matter?. BMC Public Health.

[29] ICF International. Enquête Démographique et de Santé en République Démocratique du Congo 2013–2014. Rockville, MD: Ministère du Plan et Suivi de la Mise en oeuvre de la Révolution de la Modernité, Minstère de la Santé Publique, & ICF International; 2014.

[30] Doocy S, Emerson J, Colantuoni E, Strong J, Mansen KA, The Jenga Jamaa II Study Team, et al. Improving household food security in eastern Democratic Republic of the Congo: a comparative analysis of four interventions. Food Sec. 2018;10(649-660).

[31] DataDyne. Magpi. Washington, DC. 2015.

[32] Derogatis LR, Lipman RS, Rickels K, Uhlenhuth EH, Covi L (1974). The Hopkins symptom checklist (HSCL): a self-report symptom inventory. Behav Sci.

[33] Mollica RF, Caspi-Yavin Y, Bollini P, Truong T, Tor S, Lavelle J (1992). The Harvard trauma questionnaire. Validating a cross-cultural instrument for measuring torture, trauma, and posttraumatic stress disorder in Indochinese refugees. J Nerv Ment Dis.

[34] Bass JK, Annan J, McIvor Murray S, Kaysen D, Griffiths S, Cetinoglu T (2013). Controlled trial of psychotherapy for Congolese survivors of sexual violence. N Engl J Med.

[35] Mollica RF, Wyshak G, de Marneffe D, Khuon F, Lavelle J (1987). Indochinese versions of the Hopkins symptom Checklist-25: a screening instrument for the psychiatric care of refugees. Am J Psychiatry.

[36] Lee B, Kaaya SF, Mbwambo JK, Smith-Fawzi MC, Leshabari MT (2008). Detecting depressive disorder with the Hopkins symptom Checklist-25 in Tanzania. Int J Soc Psychiatry.

[37] Rieder H, Elbert T. The relationship between organized violence, family violence and mental health: findings from a community-based survey in Muhanga. Southern Rwanda Eur J Psychotraumatol. 2013;4:10.3402/ejpt.v4i0.21329. 10.3402/ejpt.v4i0.21329 PMC3828565 24244834

[38] Foss GF, Chantal AW, Hendrickson S (2004). Maternal depression and anxiety and infant development: a comparison of foreign-born and native-born mothers. Public Health Nurs.

[39] Mollica RF, McInnes K, Sarajlic N, Lavelle J, Sarajlic I, Massagli MP (1999). Disability associated with psychiatric comorbidity and health status in Bosnian refugees living in Croatia. JAMA..

[40] Hollifield M, Warner TD, Lian N, Krakow B, Jenkins JH, Kesler J (2002). Measuring trauma and health status in refugees: a critical review. JAMA.

[41] Rasmussen A, Verkuilen J, Ho E, Fan Y (2015). Posttraumatic stress disorder among refugees: measurement invariance of Harvard trauma questionnaire scores across global regions and response patterns. Psychol Assess.

[42] Cronbach LJ (1951). Coefficient alpha and the internal structure of tests. Psychometrika.

[43] Lohman TG, Roche AF, Martorell R (1988). Anthropometric standardization reference manual.

[44] Ververs M, Antierens A, Sackl A, Staderini N, Captier V. Which anthropometric indicators identify a pregnant woman as acutely malnourished and predict adverse birth outcomes in the humanitarian context? PLoS Currents. 2013;5:ecurrents.dis.54a8b618c1bc031ea140e3f2934599c8. 10.1371/currents.dis.54a8b618c1bc031ea140e3f2934599c8 PMC3682760 23787989

[45] FAO and FHI 360. Minimum Dietary Diversity for Women: A Guide for Measurement. Rome: FAO; 2016.

[46] Coates J, Swindale A, Bilinsky P (2007). Household food insecurity access scale (HFIAS) for measurement of food access: Indicator guide (v.3).

[47] StataCorp. Stata Statistical Software: Release 13. College Station, TX: StataCorp LP; 2013.

[48] Doocy S, Emerson J, Colantouni E, Strong J, Amundson-Mansen K, Jenga Jamaa II. Study team, et al. Evaluating interventions to improve child nutrition in eastern Democratic Republic of Congo. Public Health Nutr. 2019;22(1):3-14. 10.1017/S1368980018002859 PMC10260455 30520406

[49] Tsai AC, Bangsberg DR, Frongillo EA, Hunt PW, Muzoora C, Martin JN (2012). Food insecurity, depression and the modifying role of social support among people living with HIV/AIDS in rural Uganda. Soc Sci Med.

[50] Tsai AC, Tomlinson M, Comulada WS, Rotheram-Borus MJ (2016). Food insufficiency, depression, and the modifying role of social support: evidence from a population-based, prospective cohort of pregnant women in peri-urban South Africa. Soc Sci Med.

[51] Baskin R, Hill B, Jacka FN, O'Neil A, Skouteris H (2015). The association between diet quality and mental health during the perinatal period. A systematic review. Appetite.

[52] Lai JS, Hiles S, Bisquera A, Hure AJ, McEvoy M, Attia J (2014). A systematic review and meta-analysis of dietary patterns and depression in community-dwelling adults. Am J Clin Nutr.

[53] Poorrezaeian M, Siassi F, Qorbani M, Karimi J, Koohdani F, Asayesh H (2015). Association of dietary diversity score with anxiety in women. Psychiatry Res.

[54] Johnston E, Johnson S, McLeod P, Johnston M (2004). The relation of body mass index to depressive symptoms. Can J Public Health.

[55] de Wit LM, van Straten A, van Herten M, Penninx BW, Cuijpers P (2009). Depression and body mass index, a u-shaped association. BMC Public Health.

[56] Martin-Rodriguez E, Guillen-Grima F, Auba E, Marti A, Brugos-Larumbe A (2016). Relationship between body mass index and depression in women: a 7-year prospective cohort study. The APNA Study Eur Psychiatry.

[57] Urban EJ, Charles ST, Levine LJ, Almeida DM (2018). Depression history and memory bias for specific daily emotions. PLoS One.

[58] Patten SB (2003). Recall bias and major depression lifetime prevalence. Soc Psychiatry Psychiatr Epidemiol.

