# Supplementary material for: Mental health symptoms and their relations with dietary diversity and nutritional status among mothers of young children in eastern Democratic Republic of the Congo
Source: BMC Public Health. 2020 Feb 13;20:225. doi: 10.1186/s12889-019-8092-3 (PMC7020353; doi:10.1186/s12889-019-8092-3)
Supplement: Supplementary file 1 — Additional file 1: Table 4. Associations between Independent Variables and Background Characteristics. Table 5. Associations between Dependent Variables and Background Characteristics. [file 12889_2019_8092_MOESM1_ESM.docx]

| **Table 4: Associations between Independent Variables and Background Characteristics** | | | | | | | | | |
| --- | --- | --- | --- | --- | --- | --- | --- | --- | --- |
|  | **HSCL Score^1^** | | | **HTQ Score^2^** | | | **High Distress^3^** | | |
|  | β | (95% CI) | p-value | β | (95% CI) | p-value | OR | (95% CI) | p-value |
| Fizi Territory | -0.03 | (-0.11, 0.05) | 0.486 | 0.01 | (-0.08, 0.10) | 0.856 | 0.82 | (0.58, 1.16) | 0.271 |
| Agro-ecological Zone (reference: mountains) | | | | | | | | | |
| Plains | -0.04 | (-0.17, 0.08) | 0.507 | -0.12 | (-0.25, 0.01) | 0.078 | 0.44 | (0.28, 0.69) | **<0.001** |
| Lakeside | -0.07 | (-0.20, 0.07) | 0.333 | -0.10 | (-0.24, 0.04) | 0.168 | 0.43 | (0.26, 0.71) | **0.001** |
| Household Size | 0.02 | (0.00, 0.04) | **0.018** | 0.02 | (-0.00, 0.03) | 0.067 | 1.05 | (0.98, 1.12) | 0.139 |
| Severely Food Insecure^4^ | 0.01 | (-0.08, 0.10) | 0.770 | -0.02 | (-0.12, 0.07) | 0.602 | 0.69 | (0.48, 0.99) | **0.042** |
| Intervention Group^5^ (reference: WEG) | | | | | | | | | |
| PM2A | -0.02 | (-0.14, 0.10) | 0.763 | -0.05 | (-0.17, 0.08) | 0.463 | 1.01 | (0.63, 1.61) | 0.983 |
| FFS | -0.02 | (-0.17, 0.12) | 0.759 | -0.09 | (-0.24, 0.07) | 0.275 | 1.08 | (0.61, 1.91) | 0.792 |
| F2F | -0.12 | (-0.26, 0.03) | 0.759 | -0.18 | (-0.33, -0.02) | **0.027** | 0.66 | (0.35, 1.24) | 0.194 |
| Control | -0.17 | (-0.30, -0.03) | **0.016** | -0.21 | (-0.35, -0.07) | **0.004** | 0.52 | (0.29, 0.95) | **0.034** |
| Maternal Age | 0.01 | (0.00, 0.02) | **0.006** | 0.01 | (0.00, 0.02) | **0.004** | 1.03 | (1.00, 1.06) | **0.030** |
| Maternal Education (Reference: None) | | | | | | | | | |
| Some primary school | 0.04 | (-0.05, 0.14) | 0.364 | 0.06 | (-0.04, 0.16) | 0.260 | 0.96 | (0.66, 1.39) | 0.813 |
| Some secondary school | -0.12 | (-0.24, -0.01) | **0.040** | -0.08 | (-0.21, 0.04) | 0.202 | 0.52 | (0.30, 0.90) | **0.019** |
| Married | 0.01 | (-0.13, 0.15) | 0.874 | -0.04 | (-0.18, 0.11) | 0.632 | 0.87 | (0.50, 1.52) | 0.633 |
| Pregnant | -0.02 | (-0.12, 0.09) | 0.775 | -0.08 | (-0.19, 0.03) | 0.160 | 0.70 | (0.44, 1.11) | 0.131 |
| Number of children | 0.04 | (0.02, 0.05) | **<0.001** | 0.03 | (0.01, 0.05) | **0.001** | 1.13 | (1.05, 1.21) | **0.001** |
| Living in territory of origin | -0.19 | (-0.29, -0.10) | **<0.001** | -0.20 | (-0.30, -0.10) | **<0.001** | 0.46 | (0.32, 0.66) | **<0.001** |
| Child death | 0.04 | (-0.05, 0.13) | 0.376 | 0.11 | (0.01, 0.20) | **0.029** | 1.13 | (0.78, 1.64) | 0.517 |
| Maternal self-reported health (reference: poor) | | | | | | | | | |
| Average | -0.26 | (-0.36, -0.15) | **<0.001** | -0.19 | (-0.30, -0.08) | **0.001** | 0.42 | (0.27, 0.63) | **<0.001** |
| Good | -0.34 | (-0.45, -0.24) | **<0.001** | -0.24 | (-0.35, -0.12) | **<0.001** | 0.39 | (0.25, 0.60) | **<0.001** |
| Very good/excellent | -0.66 | (-0.87, -0.45) | **<0.001** | -0.55 | (-0.78, -0.33) | **<0.001** | 0.20 | (0.06, 0.69) | **0.011** |
| ^1^24 items from the Hopkins Symptom Checklist-25 measuring depression and anxiety symptoms, range of scale scores 1-4.  ^2^16 items from the Harvard Trauma Questionnaire measuring post-traumatic stress symptoms, range of scale scores 1-4.  ^3^Participants with mean item scores in the upper quartile of both measures (≥ 2.6 for the HSCL-25 and ≥ 2.5 for the HTQ).  ^4^Household Food Insecurity Access Scale  ^5^WEG: Women’s Empowerment Group, PM2A: Prevention of Malnutrition in Children under 2 Approach, FFS: Farmer Field Schools, F2F: Farmer to Farmer.  Significant *p*-values are set in bold | | | | | | | | | |

| **Table 5: Associations between Dependent Variables and Background Characteristics** | | | | | | | | | |
| --- | --- | --- | --- | --- | --- | --- | --- | --- | --- |
|  | **Dietary Diversity Score** | | | **Body Mass Index (BMI)** | | | **Underweight^1^** | | |
|  | β | (95% CI) | p-value | β | (95% CI) | p-value | OR | (95% CI) | p-value |
| Fizi Territory | 0.22 | (0.09, 0.35) | **0.001** | 0.97 | (0.54, 1.40) | **<0.001** | 0.43 | (0.26, 0.69) | **0.001** |
| Agro-ecological Zone | | | | | | | | | |
| Plains | 0.17 | (-0.04, 0.37) | 0.105 | 0.51 | (-0.13, 1.15) | 0.119 | 0.77 | (0.43, 1.39) | 0.388 |
| Lakeside | 0.33 | (0.11, 0.54) | **0.004** | 1.20 | (0.52, 1.89) | **0.001** | 0.29 | (0.14, 0.62) | **0.001** |
| Household Size | 0.01 | (-0.01, 0.04) | 0.385 | 0.14 | (0.06, 0.23) | **0.001** | 0.94 | (0.86, 1.03) | 0.186 |
| Severely Food Insecure^2^ | -0.26 | (-0.41, -0.12) | **<0.001** | -0.57 | (-1.03, -0.11) | **0.016** | 1.14 | (0.70, 1.86) | 0.608 |
| Intervention Group^3^ | | | | | | | | | |
| PM2A | 0.20 | (0.01, 0.40) | **0.038** | -0.66 | (-1.27, -0.04) | **0.037** | 0.84 | (0.43, 1.62) | 0.595 |
| FFS | 0.15 | (-0.09, 0.39) | 0.213 | -0.61 | (-1.37, 0.14) | 0.112 | 1.53 | (0.73, 3.18) | 0.256 |
| F2F | 0.07 | (-0.17, 0.31) | 0.213 | -0.47 | (-1.23, 0.29) | 0.228 | 0.87 | (0.38, 2.00) | 0.745 |
| Control | -0.12 | (-0.33, 0.10) | 0.294 | -0.42 | (-1.12, 0.27) | 0.230 | 1.09 | (0.53, 2.23) | 0.810 |
| Maternal Age | -0.00 | (-0.01, 0.01) | 0.560 | 0.07 | (0.03, 0.10) | **<0.001** | 0.99 | (0.95, 1.02) | 0.423 |
| Maternal Education | | | | | | | | | |
| Some primary school | 0.02 | (-0.14, 0.17) | 0.823 | 0.22 | (-0.27, 0.72) | 0.371 | 1.31 | (0.77, 2.22) | 0.317 |
| Some secondary school | 0.09 | (-0.10, 0.28) | 0.366 | 0.47 | (-0.16, 1.11) | 0.142 | 1.03 | (0.52, 2.05) | 0.928 |
| Married | 0.15 | (-0.08, 0.37) | 0.204 | -0.22 | (-0.90, 0.45) | 0.517 | 1.84 | (0.72, 4.70) | 0.200 |
| Pregnant | -0.03 | (-0.20, 0.14) | 0.724 |  |  |  | 3.15 | (1.95, 5.08) | **<0.001** |
| Number of children | 0.01 | (-0.02, 0.04) | 0.647 | 0.17 | (0.08, 0.26) | **<0.001** | 0.87 | (0.79, 0.97) | **0.014** |
| Living in territory of origin | -0.26 | (-0.42, -0.11) | **0.001** | -0.14 | (-0.63, 0.35) | 0.578 | 1.74 | (0.97, 3.11) | 0.062 |
| Child death | -0.02 | (-0.17, 0.13) | 0.766 | 0.28 | (-0.20, 0.75) | 0.252 | 1.16 | (0.72, 1.87) | 0.553 |
| Maternal self-reported health | | | | | | | | | |
| Average | -0.15 | (-0.32, 0.02) | 0.078 | 0.65 | (0.11, 1.19) | **0.018** | 0.82 | (0.49, 1.39) | 0.466 |
| Good | -0.23 | (-0.41, -0.06) | **0.009** | 1.31 | (0.75, 1.87) | **<0.001** | 0.44 | (0.24, 0.82) | **0.009** |
| Very good/excellent | -0.19 | (-0.54, 0.16) | 0.291 | 1.62 | (0.51,2.73) | **0.004** | 0.43 | (0.10, 1.91) | 0.269 |
| ^1^BMI < 18.5 kg/m^2^ for non-pregnant participants, or mid-upper arm circumference < 23cm for pregnant participants.  ^2^ Household Food Insecurity Access Scale  ^3^ WEG: Women’s Empowerment Group, PM2A: Prevention of Malnutrition in Children under 2 Approach, FFS: Farmer Field Schools, F2F: Farmer to Farmer. | | | | | | | | | |

Significant *p*-values are set in bold
